# Supplementary material for: Transcription factor-promoter interactions for PSY/PSYR-mediated regulation of root growth
Source: bioRxiv. 2025 Dec 8:2025.09.29.679155. Preprint. [Version 3] doi: 10.1101/2025.09.29.679155 (PMC12642490; doi:10.1101/2025.09.29.679155)
Supplement: Supplement 1 [file NIHPP2025.09.29.679155v3-supplement-1.pdf]

bioRxiv preprint doi: https://doi.org/10.1101/2025.09.29.679155; this version posted December 8, 2025. The copyright holder for this preprint (which was not certified by peer review) is the author/funder, who has granted bioRxiv a license to display the preprint in perpetuity. It is made available under aCC-BY 4.0 International license.

Supplemental Materials

All supplemental materials referenced in the text will be provided in the final published version. All main findings are fully presented in the figures included in this manuscript.

Supplementary Figures

Supplementary Fig. 1. Overlap of eY1H and DAP-seq interactions.
Venn diagram showing TFs identified by either eY1H or DAP-seq for three *PSYR* promoters and nine *PSY* promoters. Yellow circle indicates TFs identified by eY1H, and blue circle indicates TFs identified by DAP-seq.

Supplementary Fig. 2. *PSY/PSYR* expression at the whole plant level.
Data were extracted from the publicly available Arabidopsis RNA-seq (ARS) database (http://ipf.sustech.edu.cn/pub/athrna/).

bioRxiv preprint doi: https://doi.org/10.1101/2025.09.29.679155; this version posted December 8, 2025. The copyright holder for this preprint (which was not certified by peer review) is the author/funder, who has granted bioRxiv a license to display the preprint in perpetuity. It is made available under aCC-BY 4.0 International license.

Supplementary Fig. 3. *PSY/PSYR* expression at the single-cell level in roots.
Data were extracted from the Plant sc-Atlas database.

Supplementary Fig. 4. Expression patterns of candidate TFs identified by both eY1H and DAP-seq.
Data were extracted from the publicly available bulk RNA-seq database.

Supplementary Fig. 5. Confirmation of selected TF knockouts.
qRT-PCR analysis of selected TF expression in TF knockout (KO) mutants. Gene expression was measured by qRT-PCR and normalized to wild-type (WT = 1, grey bars). Expression levels showing a significant decrease are indicated in red. Values represent mean ± SD (n = 3 biological replicates).

Supplementary Fig. 6. Comparison of primary root length in selected Arabidopsis mutant alleles of transcription factors identified by DAP-seq.
Primary root length of 10 TF KO mutants at 10 days old. Exact *P* values are reported in Supplementary Data 3.

Supplementary Fig. 7. CRF10 acts as a negative regulator of plant growth.
a. PCR genotyping confirming the presence of the *GFP-CRF10* transgene.
b. Overexpression of *CRF10* causes an extreme dwarf phenotype. Forty-day-old plants expressing GFP-CRF10 and their segregating wild-type siblings are shown. Scale bar = 1 cm.

Supplementary Datasets

Supplementary Dataset 1. List of eY1H results.

Supplementary Dataset 2. Overlap of eY1H and DAP-seq interactions.

Supplementary Dataset 3. RSA (root system architecture) phenotyping data.

Supplementary Dataset 4. qRT-PCR gene expression data.

Supplementary Tables

Supplementary Table 1. Genotyping, qRT-PCR, and cloning primers.

Supplementary Table 2. Summary of mutants, transgenic plants, and promoter-edited plants.
